# Variables Associated With Hyperkalemic Renal Tubular Acidosis in Solid Organ Transplant Recipients

**DOI:** 10.7759/cureus.55379

**Published:** 2024-03-02

**Authors:** Othmane Mohib, Julien Vanderhulst, Concetta Catalano, Ana Roussoulières, Christiane Knoop, Alain Lemoine, Thomas Baudoux

**Affiliations:** 1 Department of Nephrology, Cliniques Universitaires de Bruxelles - Hôpital (CUB) Erasme, Brussels, BEL; 2 Internal Medicine, Centre Hospitalier Universitaire (CHU) Brugmann, Brussels, BEL; 3 Department of Cardiology, Cliniques Universitaires de Bruxelles - Hôpital (CUB) Erasme, Brussels, BEL; 4 Department of Pulmonology, Cliniques Universitaires de Bruxelles - Hôpital (CUB) Erasme, Brussels, BEL

**Keywords:** tacrolimus, hypoaldosteronism, calcineurin inhibitor, renal tubular acidosis, hyperkalemia, transplantation

## Abstract

Introduction: The occurrence of hyperkalemic renal tubular acidosis (RTA) in the post-transplantation period is likely underestimated, and its identification remains important to offer adequate medical management. Transplant recipients frequently present with clinical and biological characteristics that may be associated with the occurrence of this complication.

Methods: This was a single-center retrospective study that compared transplanted patients with hyperkalemic RTA and a control group to identify variables associated with the occurrence of this complication. Fisher’s exact test and the Mann-Whitney test, followed by multivariate logistic regression, were applied to test whether there was a significant association between hyperkalemic RTA and different variables.

Results: Kidney and heart transplant recipients were at greater risk of developing RTA than lung transplant recipients (p = 0.016). There was also a significant association between the development of RTA and kalemia (p < 0.01), chloremia (p < 0.01), and bicarbonatemia (p < 0.01). The significant impact of these last three variables was confirmed by the results of the multivariate logistic regression. Residual serum tacrolimus levels (p = 0.13) and creatinine levels (p = 0.17) of renal transplant patients were not significantly associated with hyperkalemic RTA.

Conclusion: The type of transplanted organ, kalemia, chloremia, and bicarbonatemia were significantly associated with the occurrence of hyperkalemic RTA. This study calls into question certain approaches to managing this complication proposed in a number of case reports, such as reducing the target serum residual of tacrolimus or discontinuing trimethoprim-sulfamethoxazole (TMP-SMX) in favor of another antibiotic prophylactic agent, potentially exposing patients to graft rejection and opportunistic infections.

## Introduction

Solid organ transplantation requires the administration of several immunosuppressive and anti-infective drugs for the prevention of rejection and opportunistic infections. Among them, calcineurin inhibitors (CNI) - a key drug class in the prevention of rejection [1] - and trimethoprim-sulfamethoxazole (TMP-SMX) are established causes of hyperkalemic renal tubular acidosis (RTA) [2-6]. The first case of RTA that occurred after transplantation was described in 1967 [7]. Since then, all types of RTA have been reported in the post-transplant period [8,9].

RTA refers to a pattern of disorders in which, despite a preserved glomerular filtration rate, metabolic acidosis develops due to the inability of the renal tubules to maintain acid/base balance. Hyperkalemic RTA is characterized by plasmatic non-gap metabolic acidosis, hyperkalemia, and a positive urine anion gap. The urinary pH in this pathologic condition depends on the amount of buffer, particularly ammonia, present in the distal tubule lumen, but it is frequently below 5.5. There are two forms of hyperkalemic RTA: hypoaldosteronism and voltage-dependent RTA. Hypoaldosteronism is defined by aldosterone deficiency or resistance and is often referred to as “type 4 RTA.” Aldosterone deficiency may be the result of hyporeninism-hypoaldosteronism in patients with diabetes or kidney function impairment; angiotensin inhibitor use, such as angiotensin-converting enzyme inhibitors (ACEis) or angiotensin receptor blockers (ARBs); primary adrenal insufficiency; anticoagulation by heparin [10]; hereditary enzymatic deficiencies such as aldosterone synthase deficiency [11,12] or Gordon’s syndrome [13]; a severe illness condition due to adrenocorticotropic hormone (ACTH) hypersecretion, which diverts the substrate to cortisol synthesis [14]; or a transient hypoaldosteronism following surgical treatment of hyperaldosteronism [15]. Aldosterone resistance is usually iatrogenic due to the administration of potassium-sparing diuretics [16] or antibiotics such as trimethoprim [5,6] or pentamidine [17]. Rarely, aldosterone resistance may be due to a hereditary disorder such as pseudohypoaldosteronism type 1. Voltage-dependent RTA situations occur when reduced distal sodium delivery, as in severe situations of hypovolemia [18] or increased proximal tubule reabsorption, prevents the generation of an electrical gradient favorable to the secretion of potassium and hydrogen ions. Other cases of voltage-dependent RTA are related to inherited or acquired defects in the sodium transport mechanisms in the principal cell, such as in lupus nephritis [19], sickle cell disease [20], or urinary tract obstruction [21]. Whatever the form of hyperkalemic RTA, hyperkalemia is a major contributor to the development of metabolic acidosis due to the inhibition of ammonia genesis and reduction of urinary ammonium excretion [18,22]. The occurrence of hyperkalemia in the early post-transplant period is associated with significant morbidity and healthcare costs [23,24].

To date, the incidence of hyperkalemic RTA in the post-transplant period is unknown. In addition, clinical and biological variables associated with the occurrence of hyperkalemic RTA in solid organ transplant patients in the post-transplantation period have not been studied yet. Therefore, we conducted a single-center retrospective comparative study to identify these variables.

The authors would also like to mention that this article was previously posted to the Research square preprint server on April 20th, 2023.

## Materials and methods

Patient selection

We performed a single-center retrospective comparative study covering a period of five years (January 1, 2017, to January 1, 2022), using our database of kidney, heart, and lung transplant patients to identify patients with a biological profile compatible with hyperkalemic RTA. We did not have access to the database of liver transplant patients at our institution. Patients' biological data were examined from the transplantation date until June 2022. Two groups of patients were defined: those with hyperkalemic RTA and a control group. The diagnosis of hyperkalemic RTA was made based on the concomitant presence of the following four biological criteria: bicarbonatemia <23 mmol/L, kalemia >4.5 mmol/L, serum anion gap ≤20 mmol/L, and urinary anion gap >0. Urinary pH was not considered for diagnosis. The exclusion criteria for the study were as follows: proven noncompliance with drug treatment, missing clinical and biological data, or recourse to post-transplant extracorporeal renal replacement therapy (ERRT).

Qualitative and quantitative variables

The qualitative variables analyzed were the type of transplanted organ, sex, type of CNI, prophylaxis by TMP-SMX, administration of ACEi, ARB, and/or potassium-sparing diuretic, and the presence of diabetes mellitus. The quantitative variables analyzed were age, residual serum tacrolimus level, serum creatinine (Sr Cr), estimated glomerular filtration rate according to the chronic kidney disease epidemiology collaboration formula (eGFR CKD-EPI), kalemia (K), chloremia (Cl), and bicarbonatemia (HCO_3_). All quantitative variables except age were obtained 12 days (±1 day) post-transplantation in the control group, which corresponds to the median time to diagnosis of hyperkalemic RTA. The data were extracted via a standardized extraction form developed in Microsoft Excel (Microsoft® Corp., Redmond, WA).

Statistical analysis

Fisher’s exact test was applied to test whether there is a significant association between the occurrence of hyperkalemic RTA and each of the qualitative variables in the dataset. A significant threshold of α = 0.05 and a confidence interval of 95% were used. Given the small sample size, it was decided to use a Fisher exact test instead of a Chi-square test. The Mann-Whitney test was applied for the quantitative variables, with the same significance threshold and confidence interval as for the Fisher's exact test. A logistic regression, after the exclusion of variables with insufficient observations, was then applied. Creatinine and eGFR-CKD-EPI were also not included in this model because the type of transplanted organ was excluded. We were, therefore, able to obtain the full model from which we selected the variables using a stepwise procedure. The final logistic model was chosen according to the Akaike Information Criterion (AIC). A significance threshold of α = 0.05 and a confidence interval of 95% were used. The results of this logistic regression were also interpreted on the basis of odds- ratios (OR). The statistical analysis was reviewed by an experienced senior statistician.

## Results

**Figure 1:**
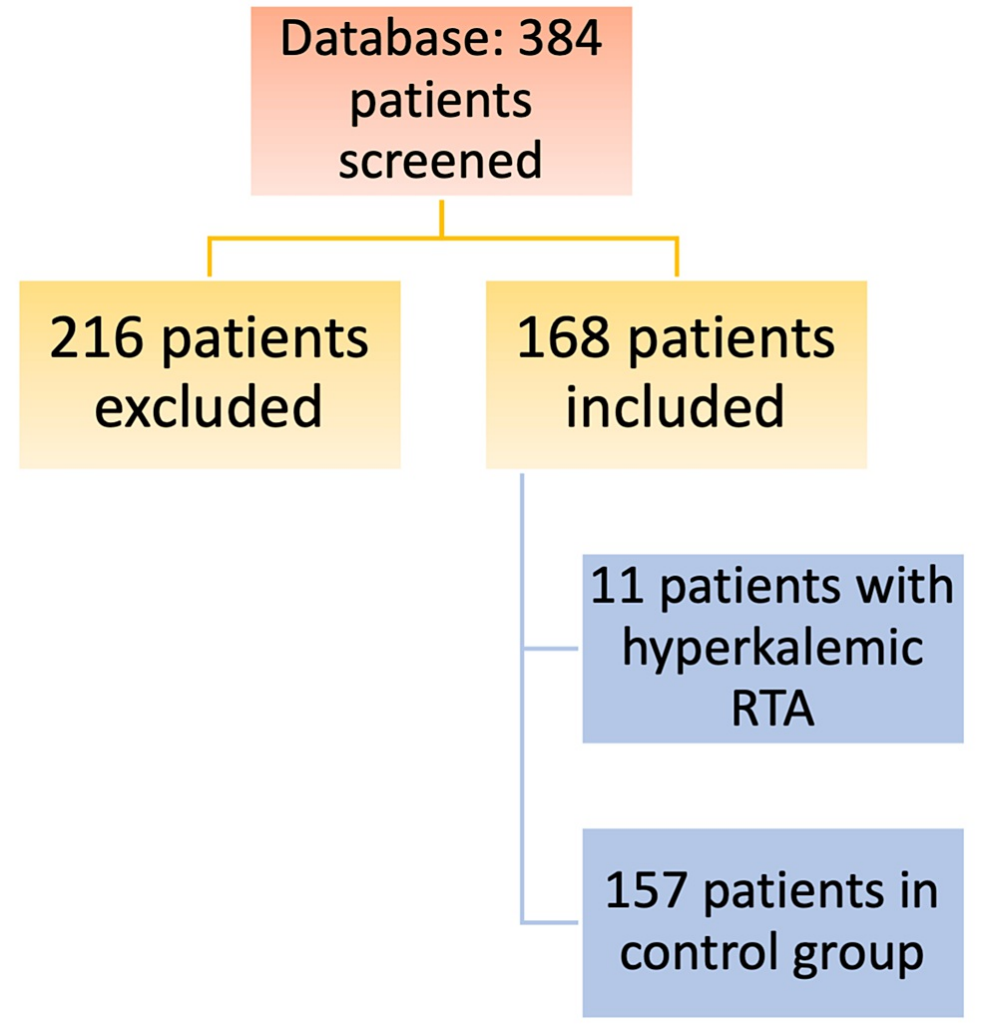
Flow chart of study population RTA: renal tubular acidosis

A total of 384 patient records were analyzed. Among these, 216 patients were excluded from the study (34 patients for use of ERRT and 182 patients for missing data or early death). Finally, 168 patients were included in the study. A flow chart of the study population is shown in Figure 1. Among the included patients, 11 cases of hyperkalemic RTA were identified, along with 157 controls.

The incidence of hyperkalemic RTA was 6.5% among the included records. All diagnoses of hyperkalemic RTA were made retrospectively. In the hyperkalemic RTA group, the median time from transplantation to diagnosis was 12 days. The earliest diagnosis of hyperkalemic RTA was made within three days of transplantation, and the latest was made 42 days after transplantation.

Details of the qualitative variables for the two groups are shown in Table 1. No diagnoses of hyperkalemic RTA were made in the lung transplant subgroup (n = 59). Most patients in the hyperkalemic RTA group were kidney transplant recipients (8/11) and/or males (9/11), and all patients in this group were treated with TMP-SMX (11/11). Very few of the patients were administered cyclosporine (n = 7), regardless of the transplanted organ. Moreover, there were no cases of hyperkalemic RTA with cyclosporine. Regarding ACEi, ARB, and potassium-sparing diuretics, these therapies were only rarely prescribed in the immediate post-transplant period, and there were no occurrences of hyperkalemia associated with these treatments. Finally, among the 45 diabetic patients included in the study, three developed hyperkalemic RTA. When the qualitative variables of the dataset were evaluated using Fisher’s exact test, the type of transplanted organ was the only qualitative variable significantly associated with the occurrence of hyperkalemic RTA (p=0.016). Patients who received a heart or kidney transplant were more at risk of developing hyperkalemic RTA than those who received a lung transplant.

**Table 1:** Analysis of qualitative variables in patients who developed hyperkalemic RTA and controls ACEi: angiotensin-converting enzyme inhibitor; ARB: angiotensin receptor blocker; CNI: calcineurin inhibitor; RTA: renal tubular acidosis; TMP-SMX: trimethoprim-sulfamethoxazole.

Qualitative variables		Hyperkalemic RTA group (n=11)	Control group (n=157)	p-value
Transplanted organ	Heart	3 (27%)	24 (15%)	0.016
	Kidney	8 (73%)	74 (47%)	
	Lung	0	59 (38%)	
Sex	Female	2 (18%)	63 (40%)	0.21
	Male	9 (82%)	94 (60%)	
CNI type	Cyclosporine	0	7 (5%)	1
	Tacrolimus	11 (100%)	150 (95%)	
Other drugs	TMP-SMX	11 (100%)	144 (92%)	1
	ACEi	0	9 (6%)	1
	ARB	0	1 (0.6%)	1
	Potassium-sparing diuretics	0	3 (2%)	1
Diabetes mellitus		3 (27%)	42 (27%)	1

**Figure 2:**
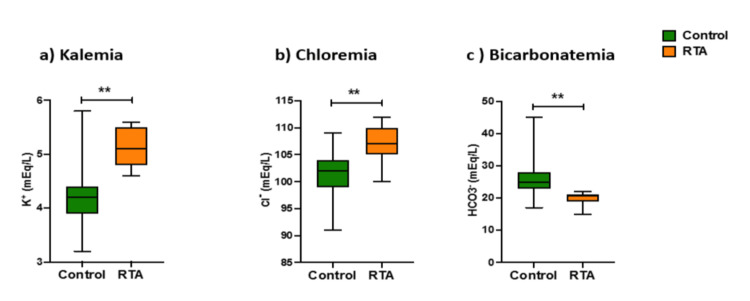
Box-plots (median; min-max) of kalemia (a), chloremia (b), and bicarbonatemia (c) within the control and RTA groups *p<0.05 ; **p<0.01. RTA: renal tubular acidosis.

**Figure 3:**
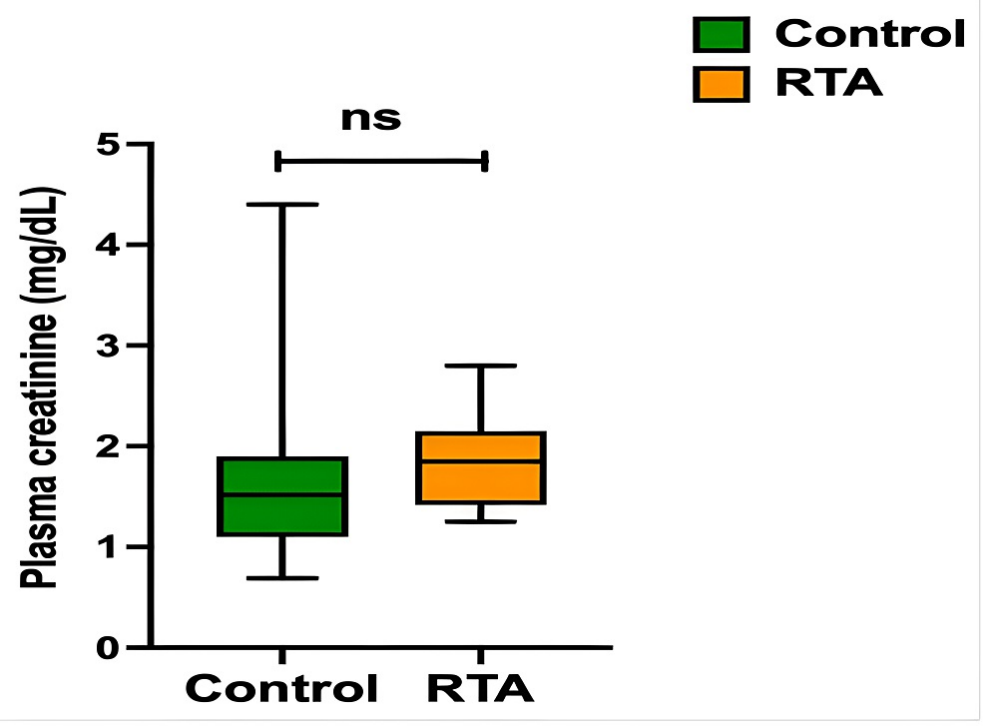
Box-plots (median; min-max) of creatininemia within the control and RTA subgroups of kidney transplant recipients ns: not significant; RTA: renal tubular acidosis

Data from the analysis of the quantitative variables in both groups is shown in Table 2. The variables were analyzed using the Mann-Whitney test. A significant difference between the two groups was observed for the following variables: K, Cl, and HCO_3_. Box plots for these variables are presented in Figure 2. To avoid introducing interpretation bias, creatinine levels for kidney transplant recipients were compared separately from the other subjects due to their higher post-transplant creatinine levels. Sr Cr was not significantly associated with the occurrence of hyperkalemic RTA (p=0.17) (Figure 3), and neither were serum residual tacrolimus levels (p=0.13) or age (p=0.96).

**Table 2:** Analysis of quantitative variables in patients who developed hyperkalemic RTA and controls CKD-EPI: chronic kidney disease epidemiology collaboration; Cl: chloremia; eGFR: estimated glomerular filtration rate; HCO_3_: bicarbonatemia; K: kalemia; Sr Cr: serum creatinine.

Variables	Hyperkalemic RTA group		Control group		p-value
	Mean	Median (min-max)	Mean	Median (min-max)	
Age (years)	51.55	57.00 (26–70)	51.85	56.00 (18–74)	0.96
Cl (mmol/L)	107	107 (100–112)	101.38	102.00 (91–109)	<0.01
GFRe CKD-EPI (ml/mn/1.73 m^2^)	42.36	45 (23–62)	77.92	77.00 (15–159)	NA
HCO_3_ (mmol/L)	20	21.00 (15–22)	25.38	25.00 (17–45)	<0.01
K (mmol/L)	5.14	5.10 (4.6–5.6)	4.19	4.20 (3.2–5.8)	<0.01
Sr Cr (mg/dL)	1.82	1.74 (1.25–2.8)	1.19	1.00 (0.34–4.4)	NA
Residual cyclosporine (ng/mL)	N/A	N/A	157.00	180.00 (79–255)	NA
Residual tacrolimus (ng/mL)	9.56	10.10 (6.2–11.5)	8.85	8.25 (2.4–23.2)	0.13

Multivariate logistic regression was performed, excluding variables with levels without observations as well as the residual serum tacrolimus level because there were not enough observations for the model to converge. Sr Cr and eGFR-CKD-EPI were also not included in this model because the type of transplanted organ was excluded (there were not enough observations and no interest in combining them). The full model was obtained, from which the variables were selected using a stepwise procedure. The best logistic model, according to the AIC, was the final model. The results of this multivariate logistic regression are presented in Table 3. The conclusion of this multivariate logistic regression was that each of the following quantitative variables - K, Cl, and HCO_3_ - was associated with the occurrence of hyperkalemic RTA while considering the other quantitative variables previously mentioned, as well as age, sex, and the presence of diabetes. When interpreting the results of the final model of this multivariate logistic regression as ORs, it was noted that, for example, an additional unit of K increased the risk of developing hyperkalemic RTA by a factor of 303.21.

**Table 3:** Results of the multivariate logistic regression analysis Cl: chloremia; HCO_3_: bicarbonatemia; K: kalemia; OR: odds ratio; RTA: renal tubular acidosis

Variable	Control group n or mean	Hyperkalemic RTA group n or mean	OR (full model)	OR (final model)
Age	51.9	51.5	1.00 (0.90–1.11, p=0.999)	-
Gender female	63	2	-	-
Gender male	94	9	5.14 (0.08–3632.42, p=0.513)	-
K	4.2	5.1	607.30 (16.12–583894.09, p=0.011)	303.22 (17.20–31860.32, p=0.002)
Cl	101.4	107.0	2.29 (1.41–5.63, p=0.010)	2.16 (1.37–4.32, p=0.007)
HCO_3_	25.4	20	0.52 (0.27–0.81, p=0.014)	0.53 (0.29–0.82, p=0.013)
Diabetes (no)	115	8	-	-
Diabetes (yes)	42	3	4.25 (0.18–199.73, p=0.389)	-

## Discussion

To the best of our knowledge, this is the first study to identify the clinical and biological variables associated with the occurrence of hyperkalemic RTA in solid organ transplant recipients. First, this study reports an incidence of 6.5% of hyperkalemic RTA in this retrospective setting, suggesting that the incidence of this disease should not be minimized. Second, the data presented here suggest that heart and kidney transplant recipients may be at greater risk of developing hyperkalemic RTA than lung transplant recipients. This is consistent with other reports in the literature. Indeed, to date, there have been no reported cases of hyperkalemic RTA in lung transplant recipients. Our hypothesis is that the metabolic alkalosis that compensates for the frequent respiratory acidosis in end-stage respiratory failure must play a protective role, with perhaps hyperactivity of alpha-intercalated cells in the collecting tubes of these patients.

Renal dysfunction is an established risk factor for the occurrence of hyperkalemic RTA. Indeed, the volume overload encountered during renal dysfunction stimulates the secretion of atrial natriuretic peptide (ANP), which has a negative effect on both renin and aldosterone secretion induced by hyperkalemia - resulting in hyporeninism - hypoaldosteronism [25]. However, no significant plasma creatinine increase was observed in the RTA subgroup of renal transplant recipients as compared to the control subgroup of renal transplant recipients in our study.

Hyperkalemic RTA has been described in association with both cyclosporine and tacrolimus treatment in many case reports [26-29]. We did not find any data in the literature comparing the risk of the occurrence of hyperkalemic RTA between these two molecules. In this study, the type of CNI did not seem to impact the occurrence of hyperkalemic RTA. However, the number of patients on cyclosporine included was very low, complicating the interpretation. This very low proportion of patients on cyclosporine could be explained by the fact that tacrolimus is often preferred to cyclosporine by clinicians, based on multiple meta-analyses and randomized trials that indicate a lower rate of rejection regardless of the type of transplanted organ (heart, liver, kidney, or lung) [30-33]. CNIs may lead to this complication by inducing hyporeninism-hypoaldosteronism, decreasing mineralocorticoid receptor expression by downregulation, and mimicking pseudohypoaldosteronism type 2 by stimulating the activity of the thiazide-sensitive renal sodium chloride co-transporter in the distal convoluted tubule [34-37]. In addition, in this study, residual serum tacrolimus levels were not associated with the occurrence of hyperkalemic RTA. However, in an observational study of 106 kidney transplant patients, serum tacrolimus levels appeared to be an independent risk factor for RTA (all types) [38]. Even if the occurrence of hyperkalemic RTA is attributed to CNI, its discontinuation is often problematic in the immediate post-transplant period. Lin et al. reported on the efficacy of tacrolimus dose reduction for the resolution of hyperkalemic RTA with the sometimes early introduction of everolimus [29]. However, it remains to be proven that the occurrence of hyperkalemic RTA associated with CNIs is dose-dependent, which is in contradiction with our results based on the comparison of residual tacrolimus levels. In addition, this decrease in CNI dosage could expose patients to a risk of rejection and, therefore, graft dysfunction. Belatacept and mammalian target of rapamycin (mTOR) inhibitors offer an alternative to CNIs in the event of hyperkalemia; however, they should be prescribed to the appropriate patient.

Concerning antibioprophylaxis for Pneumocystis jirovecii, on the basis of our results, it does not seem justified to us to propose an antibioprophylactic agent other than TMP-SMX as our first choice in order to reduce the risk of the occurrence of hyperkalemic RTA. TMP-SMX is the most effective preventive treatment for P. jirovecii [39]. However, alternative agents have been studied and can be used instead of TMP-SMX if the patient develops hyperkalemic RTA. Except for pentamidine, which has an “amiloride-like” effect by inhibiting the epithelial sodium channel (ENaC) and inducing resistance to aldosterone [17], dapsone and atovaquone are treatments of choice. 

Renin-angiotensin-aldosterone system (RAAS) blockers or potassium-sparing diuretics are rarely prescribed in the early post-transplant period, regardless of the organ transplanted. They are even usually avoided in the early post-transplantation period in renal transplant patients because of their adverse hemodynamic effect on renal filtration. This probably explains, in part, the absence in the literature of associations between the use of one of these medications and the occurrence of hyperkalemic RTA in the post-transplant period. Analysis of our small sample of transplant recipients receiving a RAAS blocker or potassium-sparing diuretic did not show an association between these medications and the occurrence of hyperkalemic RTA.

Diabetes is a well-known cause of hyporeninism-hypoaldosteronism because of defective conversion of prorenin to renin [40]. However, we did not demonstrate a significant association between diabetes mellitus and hyperkalemic RTA in this study. 

As initially suspected, hyperkalemia, hyperchloremia, and metabolic acidosis were significantly associated with a risk of developing hyperkalemic RTA. Importantly, in this study, the OR analysis of the final multivariate logistic regression model concluded that a 1-unit increase in kalemia increased the risk of hyperkalemic RTA by 300 times, and, thus, the occurrence of hyperkalemia should prompt the clinician to be vigilant.

This study had two main limitations. The first was the small sample of patients. However, while waiting for larger studies, this is the first comparative study with a control group interested in identifying the clinical and biological criteria predisposing to the occurrence of hyperkalemic RTA in solid organ transplant recipients. The second limitation was the monocentric design of the study.

## Conclusions

In this study, the type of transplanted organ and the presence of kalemia, chloremia, or bicarbonatemia were significantly associated with the occurrence of hyperkalemic RTA. It is essential to identify this complication and treat hyperkalemia first before proposing additional treatments. This study calls into question certain approaches to managing this complication proposed in a number of case reports, such as reducing the target serum residual of tacrolimus or discontinuing TMP-SMX in favor of another antibioprophylactic agent, potentially exposing patients to the risk of graft rejection and opportunistic infections. Larger studies are needed to more accurately define transplanted patients at risk for hyperkalemic RTA, notably genetic factors. Indeed, the activity or sensitivity of RAAS to various clinical or biological factors may also be involved.
